# Influence of future climate scenarios using CMIP 5 data on malaria transmission in India

**DOI:** 10.1186/s12936-024-05129-0

**Published:** 2024-10-09

**Authors:** Subrahmanya Hari Prasad Peri

**Affiliations:** Centre for Ocean, River, Atmosphere and Land Sciences, Indian Institute of Technology Kharagpur, Kharagpur, 721 302 India

**Keywords:** Malaria risk, Climate change, VECTRI model, CMIP5 data, EIR

## Abstract

**Background:**

Vector-borne diseases, such as malaria, pose a significant global threat, and climatological factors greatly influence their intensity. Tropical countries, like India, are particularly vulnerable to such diseases, making accurate estimation of malaria risk crucial.

**Methods:**

This study utilized the well-known Vector-borne Disease Community Model, VECTRI, developed by the International Centre for Theoretical Physics in Trieste. The model was implemented to estimate malaria’s Entomological Inoculation Rate (EIR). Future climatic prediction datasets, including CMIP 5 and population data sets, were used as inputs for the analysis. Three RCP scenarios are considered (Representative Concentration Pathways are climate change scenarios that project radiative forcing to 2100 due to future greenhouse gas concentrations). The projections covered the period from 1 Jan, 2020, to 31 Dec, 2029.

**Results:**

The estimated mean EIR for the years 2020–2029 ranged, and a significant decline in malaria risk was observed with all RCP 2.6, 4.5, and 8.5 scenarios. Each year 0.3 to 2.6 [min–max] EIR/person/day decline is observed with a strong decline in man rainfall ranging from 5 to 17 [min–max] mm/year and associated high temperatures ranging from 0.03 to 0.06 [min–max] °C/year. During the post-monsoon period, August to November were identified as highly prone to malaria transmission. Spatial analysis revealed that the east coast of India faced a higher vulnerability to malaria risk, which kept increasing through RCP scenarios. Thus, it is essential to exercise caution, especially in areas with heavy rainfall.

**Conclusion:**

This research provides valuable insights for policy-makers, highlighting the need to implement future strategies to mitigate malaria risk effectively. By utilizing these findings, appropriate measures can be taken to combat the threat posed by malaria and protect public health.

## Background

The vector-borne diseases such as malaria are significantly associated with the local meteorological conditions. The basic life cycle of a mosquito depends on both humans and climate. Anopheles mosquitoes lay their eggs in various types of freshwater or brackish water, exhibiting species-specific preferences. The eggs typically hatch within a few days, and the resulting larvae undergo a developmental period of 9–12 days to reach adulthood in tropical regions [1]. However, if the larval habitats dry up prematurely, the larvae perish. Conversely, excessive rainfall can flush them away, leading to their destruction. The survival of mosquito larvae is uncertain, and the majority do not make it to adulthood [2].

The lifespan of adult mosquitoes is also relatively brief, influenced by temperature and humidity levels. Only older female mosquitoes are capable of transmitting malaria, as they must live long enough for sporozoites to develop and migrate to their salivary glands. According to the Center for Disease Control and Prevention, this development process requires a minimum of nine days under warm temperatures (around 30 °C), while it takes even longer in cooler climates. When temperatures drop below a certain threshold (15 °C for *Plasmodium vivax* and 20 °C for *Plasmodium falciparum*) [3, 4], the development cannot reach completion, and malaria transmission becomes impossible. Consequently, malaria transmission is more prevalent in warm and humid regions, whereas in temperate areas, transmission is limited to the hot days.

The proper temperatures (i.e. temperature ranging from 20–30 °C) and land cover with enough clear water availability is needed to achieve the aforementioned process. At present, there is a rapid climate change and associated elevated temperatures and extreme precipitations over the sub-continent of India [5]. This may be the best habitable environment for parasites, resulting in a surge in malarial outbreaks in future scenarios. Though parasite transmission depends on meteorological parameters, the relationships between weather, climate, and *Anopheles* habitat suitability are complex and not necessarily linear. The rainfall may create a temporary water body (site for reproduction) where the malaria vector grows and develops. However, the flushing of eggs due to extreme rainfall increases the mortality [6] of early-stage larvae, thus resulting in the destruction of malaria vectors. Thus, future projections of climate and parasite transmission are highly needed. This can be achieved by dynamic models, such as the Liverpool Malaria Model (LMM) [7], Mapping Malaria Risk in Africa (MARA) [8], Modelling Framework for the Health Impact Assessment of Man-Induced Atmospheric Changes (MIASMA) [9], and Vector-borne Disease Community Model of Centre for Theoretical Physics Trieste (VECTRI), but to, achieve this goal, in present study most resent and highly adaptive Vector-borne Disease Community Model of International Centre for Theoretical Physics Trieste (VECTRI) [10] and estimated the entomological inoculation rate of malaria (EIR) is chosen.

In this paper, the possible impact of future climate scenarios on EIR in India is assessed using a combination of epidemiological data and CMIP5 climate predictions in the VECTRI modelling framework. Scenarios from lowest to highest possible emissions (Representative Concentration Pathways 2.6, 4.5, 8.5) are assessed in the model.

## Methods

### Study area data

**Fig. 1:**
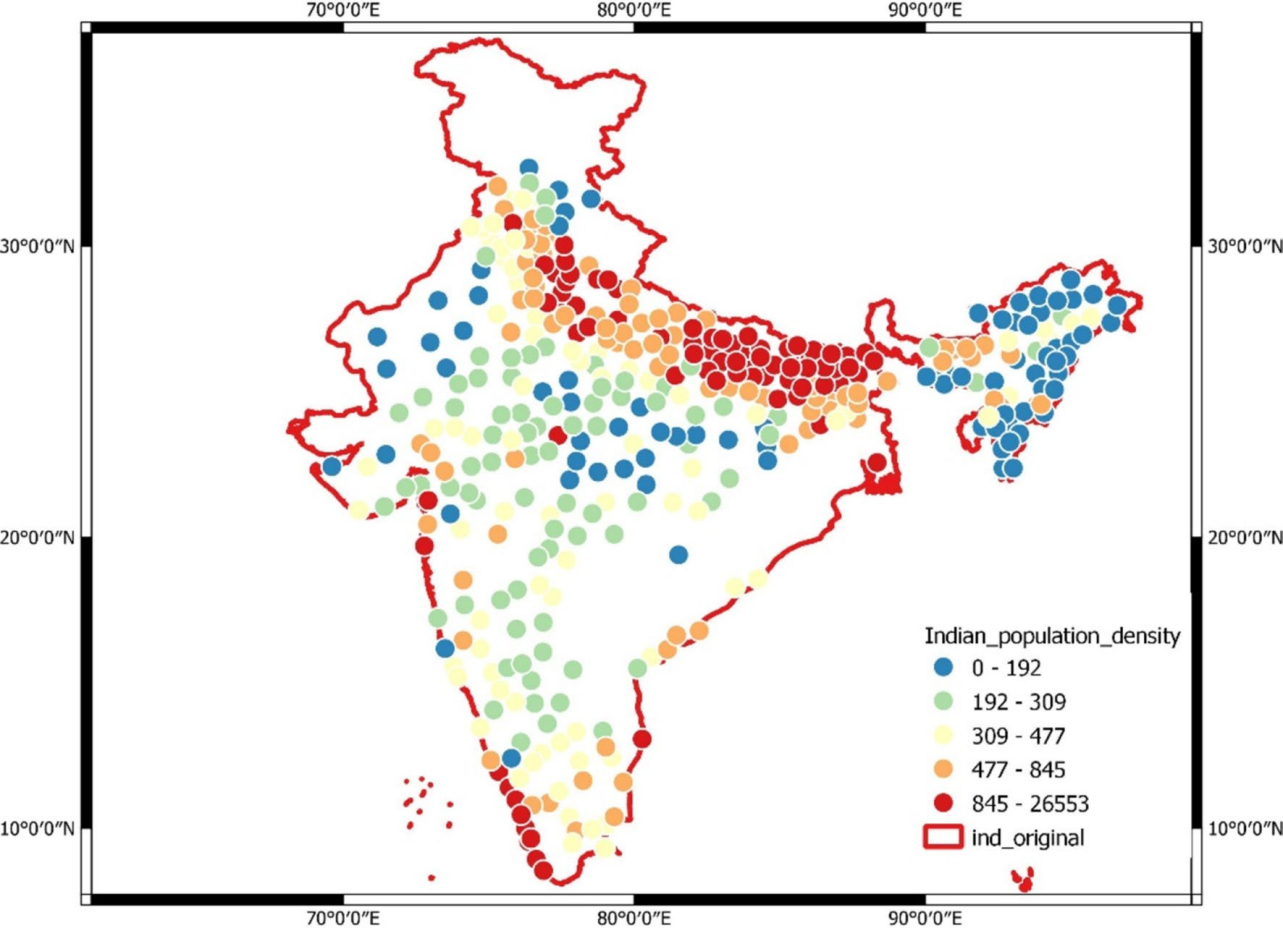
Discrete population density used in study

Indian mainland is almost extended to 8° 4ʹ north to 37° 6ʹ north latitude and 68° 7ʹ east to 97° 25ʹ east longitude (Fig. 1). India’s climate is indeed remarkably diverse. According to the Köppen system, it encompasses six major climatic subtypes:

Desert climate (BWh and BWk): In the western regions, India experiences arid and semi-arid conditions. The Thar Desert in the northwest falls into this category.

Highland climate: The northern Himalayan regions exhibit highland climates, including sub-arctic, tundra, and ice cap conditions. These vary with elevation and are characterized by cold temperatures.

Subtropical climate*:* The northern lowlands, such as Srinagar, have subtropical conditions. Some areas at higher altitudes touch continental climates.

Tropical climate: Much of the south and east of India exhibit tropical climate conditions. These regions support lush rainforests due to their warm and humid environment.

Monsoonal regime: India’s geography, including the Thar Desert and the Himalayas, plays a crucial role in its monsoonal regime. The Himalayas block frigid winds from the Tibetan Plateau, keeping North India warm during winter and hot during summer. South India tends to be warmer and more humid due to its coastlines.

Microclimates: India’s diverse topography results in starkly different microclimates, contributing to its status as one of the most climatically varied countries globally, in which Tropical, subtropical, semi-arid, and temperate zones are vulnerable to malaria risk. In India there, Most Indian regions, such as eastern states such as Chhattisgarh, Odisha, and Jharkhand, as well as the northeastern states including Tripura, Assam, Meghalaya, and Manipur, are the best habitable environment for malaria vectors” or “best environment for malaria transmission” [11]. However, due to climate change, these regions are shifting throughout India [12]. Thus, the VECTRI model, which includes both climate and population density, is useful in predicting malaria transmission.

Since the model relies on epidemiological and climatic data, the weather data was collected from the Copernicus ECMWF data portal [13]. The data is CMIP5 model-derived data with the lowest possible to highest emission scenario of Representative Concentration Pathways RCP 2.5, 4.5 and 8.5. The data comprises the daily mean of temperature and rainfall over India with 1.875° × 1.85° and rescaled to 0.35° × 0.35° for a better understanding of spatial patterns. This smoothing process was done using a linear interpolation technique from Python libraries called Scipy, and an interp function was used.

Here, RCP future climatic data is a prediction from weather prediction models. The details of the data are presented in Table 1. The VECTRI model also uses population density, which is taken from [14]; this data is real-time observations given by the Government of India. Some empirical data that are useful for running the model for Anopheles development are taken from previous studies, as stated in the Table 2.

**Table 1:** Climatic future projection of CMIP 5 data summary

Experiment:	Representative Concentration Pathway (RCP) 2.6, 4.5, 8.5 [34]
Variable:	2m temperature, Mean precipitation flux
Model:	Max Planck Institute Earth System Model MPI-ESM-LR (MPI, Germany)
Ensemble member:	r1i1p1

**Table 2:** VECTRI model parameters

Description	Value	Unit
Eggs laid per female vector	80 [35]	–
Maximum temperature for larvae survival	34 [36]	°C
Minimum temperature for larvae survival	18 [36]	°C
Time for egg hatching	1 [37]	Days
Time for pupae stages	4 [38]	Days
Minimal daily survival L1 larvae after intense rainfall	0.4	–
Exponential decay of flushing with rain rate	20 model parameter, default value is considered	mm day^−1^
Threshold temperature for egg development in vector	7.7 There’s no singular figure universally applicable to all vector species. However, studies often discuss temperature-dependent development rates in mosquitoes, thus default model value is considered	°C
Degree days for egg development in vector	37.1	Days
Threshold temperature for parasite development	16 [20]	°C
Degree days for parasite development	111 [38]	Days
Fraction of population renewed each year	0.02	–
Minimum anthropophilic biting rate	0.1	–

### Methods

In the present study, the VECTRI dynamical malaria model, developed by the International Centre for Theoretical Physics (ICTP), is utilized. This mathematical-biological model takes into account two crucial meteorological parameters—temperature and rainfall—as well as population density to assess malaria transmission. The VECTRI model involves solving a set of equations that capture the life cycles of key malaria vectors. In India, malaria is predominantly caused by two parasites, *Plasmodium falciparum* and *Plasmodium vivax*. Approximately 75% of all malaria cases in India are attributed to *P. falciparum,* while *P. vivax* accounts for a smaller portion of prevalence in some Indian states. The VECTRI setup considers the climatic sensitivity of the life cycle processes of the *Anopheles* Cruciferae vector and the *P. falciparum* parasite.

*Anopheles* mosquitoes undergo a progression from the egg stage to the larval and pupae stages, ultimately leading to the emergence of adult mosquitoes, contingent upon favourable environmental conditions. The VECTRI model incorporates the concept of degree days to represent the completion of both the sporogonic cycle and the gonotrophic cycle of the mosquito. Notably, the VECTRI treats the host and host-vector interaction as a single system, rather than as separate entities.

Regarding rainfall, the VECTRI model parameterizes its effect based on surface hydrology. At lower rainfall values (measured in mm/day), larval development takes place up to a certain threshold (7–8 mm/day). Beyond this threshold, a flushing phenomenon occurs, which provides the necessary aquatic environment for oviposition and the development of the aquatic stages of the mosquito. The volume of water in temporary water bodies, such as ponds serving as breeding sites, critically depends on factors like infiltration, evaporation, and overflow. The model considers an evaporation loss of water at 5 mm/day, which is relatively minimal compared to infiltration and overflow rates. Additionally, the VECTRI model includes a latent heat flux of 145 Wm^−2^ and sets the infiltration rate at 245 mm/day.

Population density influences the biting rate and transmission probabilities of mosquitoes. In the VECTRI model, the parameters are customized to suit the specific context of India and are outlined in Table 2.

In summary, the VECTRI model used in the study considers the impact of temperature, rainfall, and population density on malaria transmission. It stimulates the life cycles of malaria vectors and parasites while taking into account various climatic factors and their influence on the prevalence of malaria in India.

This malaria transmission model considers how local climate and precipitation affect the larval and adult life cycles of the malaria vector as well as the pathogen itself. This model also accounts for human population and land cover dynamics. The hot monsoon days of the Indian climate has a huge socio-economic impact [15, 16] in case of a surge in malaria transmission. Thus, the study aimed to analyse and predict future malaria transmission through the estimation of EIR with different climatic scenarios. This estimation of EIR was modelled for *Anopheles culicifacies*, even though some regions have *Anopheles fluviatilis*, but major contribution is due to (> 70%) *An. culicifacies* [17]

### Description of the new VECTRI model

The VECTRI model is a dynamical model for malaria transmission with grid cell distribution. Developed by the International Centre for Theoretical Physics (ICTP), it focuses on vector-borne diseases. Here are some key points about VECTRI:

1. Physically Based Treatment of Surface Hydrology: VECTRI incorporates surface hydrology with simple physical model. This allows it to account for factors like rainfall and water availability, which influence mosquito breeding sites.
2. Population Density Consideration: When calculating biting rates and transmission probabilities, VECTRI takes into account population density. This feature is crucial because it has ability to distinguish between rural and peri-urban areas of transmission rates.
3. Dynamic Framework for Development: VECTRI’s link to population density allows for active development. It can incorporate factors such as immunity, migration, socio-economic status, urbanization, and interventions. This flexibility makes it suitable for regional or even continental-wide simulations.
4. Resolution and Novel Aspects: VECTRI can be resolved with a fine spatial resolution up to 10 km or less. It explicitly models the growth stages of the egg-larvae-pupa cycle, as well as the gonotrophic and sporogonic cycles in mosquitoes.

### RCP scenarios description

Representative Concentration Pathways (RCPs) are climate change scenarios that project radiative forcing to 2100 due to future greenhouse gas concentrations. RCPs are named based on their total radiative forcing by or after 2100. Radiative forcing is a measure of the additional energy taken up by the Earth system due to increases in climate change pollution. Positive radiative forcing means the planet warms, and negative radiative forcing means the planet cools.

#### RCP 4.5

The Intergovernmental Panel on Climate Change (IPCC) describes this as a moderate scenario where emissions peak around 2040 and then decline. For the same time period as RCP 8.5, RCP 4.5 would mean an increase of 4.5 watts per square metre (W/m^2^).

#### RCP 8.5

This is a high-emission scenario with a rise in radiative forcing to 8.5 W/m in 2100.

#### RCP2.6

This represents a pathway where greenhouse gas emissions are strongly reduced, resulting in a best-estimated global average temperature rise of 1.6 °C by 2100 compared to the pre-industrial period.

- These RCP data were collected from CMIP5 models. Thus, bias might exist; for more details, please refer [18]. Since the CMIP5 data was generated from GCM (General circulation models) which may have different biases.
- Cascading mean-state biases: These can lead to overly wet solutions. They can be caused by unstable lower troposphere, cold troposphere, and zonal wind speed biases. Radiation biases: These can be caused by clouds and other geophysical variables. Many GCMs have a cold air temperature bias and a moist tropospheric humidity bias. Errors due to limited spatial resolution: These can be caused by large grid sizes. Errors due to simplified physics: These can be caused by simplified thermodynamic processes and physics.
- However, these biases will have only marginal difference in VECTRI model because it has been studied earlier in research like [19, 20].

### Step-wise implementation of VECTRI model

1. Installing the VECTRI model
2. Download RCP daily temperature and daily precipitation data
3. Converting precipitation data to mm/grid/day and temperature from Kelvin to degree centigrade.
4. Renaming the precipitation data to rain and concat precipitation and temperature data to a single NetCDF file.
5. Downloading population data and making a population flux to single NetCDF data with corresponding lat long data.
6. Running a series of simulations and obtaining daily EIR data per grid cell.

### Regression analysis

To analyse the malaria risk, true incidence data sets were chosen from the National Center for Vector Borne Diseases Control (NCVBDC) [21] and fitted the regression models of three different scenarios (RCP 2.6, RCP 4.5, and RCP 8.5) of historical simulations (2010–2019).

Here, the dependent variable is the number of *P. falciparum* cases in the whole country, which are retrieved from the Center for Vector Borne Diseases Control (NCVBDC) [21] and the independent variables are annual mean EIR, which is estimated from the VECTRI model with RCP 2.6, RCP 4.5, and RCP 8.5, and year corresponding to cases observed also taken to analyse the trend. A simple OLS (ordinary least square regression) model is considered.

$$Pf_{{\left( {annual_{c} \;ases} \right)}} = a \times EIR + b \times Year + C$$

Here, a is the slope of EIR, and b is the annual increasing/decreasing cases trend per year, and C is the intercept.

## Results

The VECTRI model was run with climatic data from 01-01-2020 to 31-12-2029 and population density from Indian census data (2011). The results are presented with annual, monthly, and spatial trends to understand the dynamics of malaria.

### Annual trends of EIR and climatological variables with different RCP scenarios

**Fig. 2:**
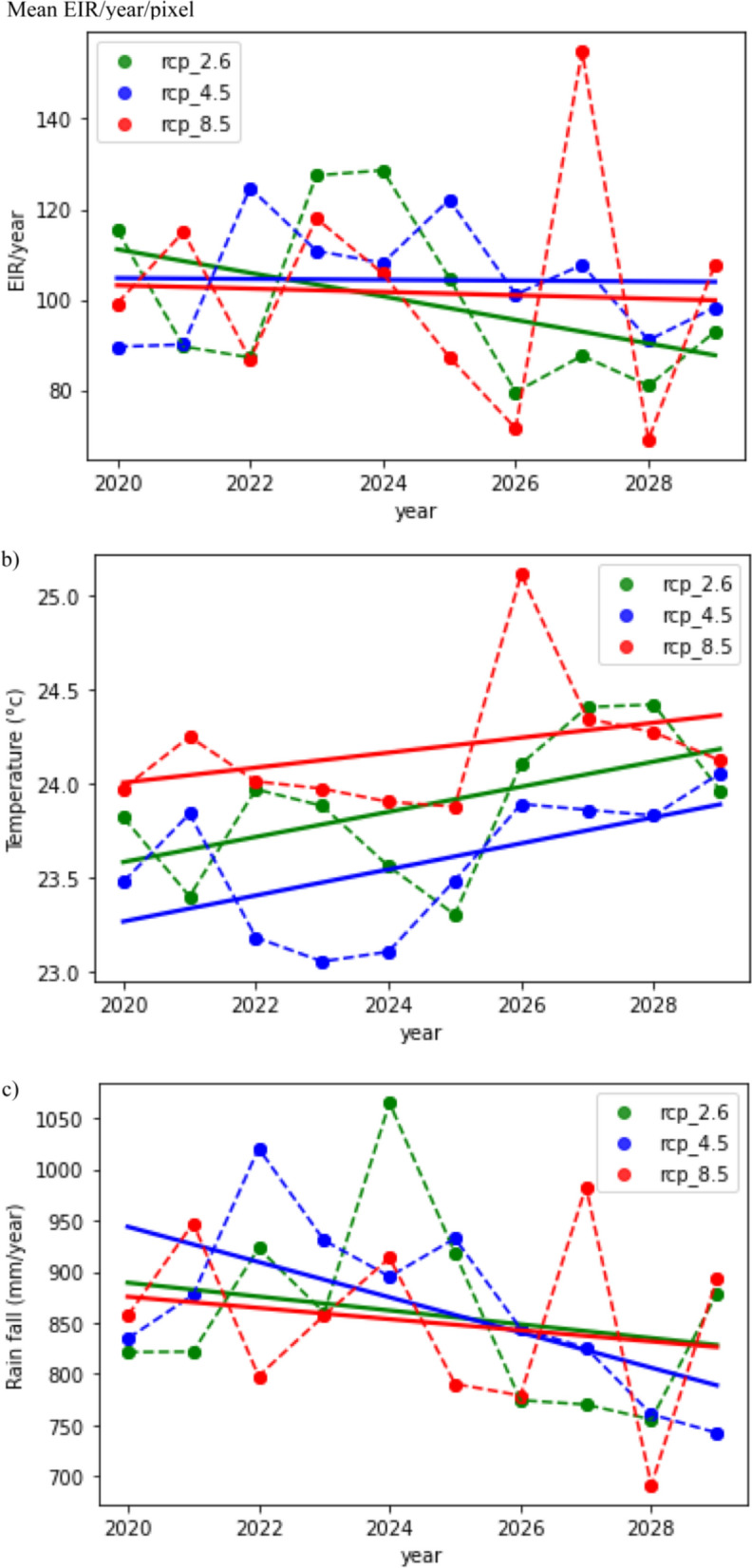
Annual trends of EIR, temperature and precipitation

The study’s key findings indicate an annual decreasing trend in the Entomological Inoculation Rate (EIR) across India. As shown in Fig. 2a, the average infectious biting has decreased over the analysed period in all three emission cases RCP 2.6, 4.5, and 8.5. Though they have a decreasing trend, the highest decreasing slope is with RCP 8.5 (− 2.6036), and the lowest is with RCP 4.5 (− 0.3669).

At the same time, there is an observed increasing trend in temperature (Fig. 2b) and a slight decreasing trend in rainfall (Fig. 2c). Though the rainfall has a decreasing trend, the highest decreasing slope is with RCP 8.5 (− 5.4681), and the lowest is with RCP 4.5 (− 17.211).

The temperature shows a positive trend of approximately 0.03–0.06 °C per year, while there is a downward trend in rainfall at a rate of about − 5.4 to − 17.21 mm per year. Temperatures have an increasing trend; the highest decreasing slope is with RCP 4.5 (0.0667), and the lowest is with RCP 8.5 (0.0398).

These results strongly suggest there is a decrement in EIR and this is a positive sign from an epidemiological perspective. It’s worth noting that there is also a noticeable surge in EIR, as seen in Fig. 2a and c, which is predicted to occur in 2027 (as per RCP 8.5) and 2024 (as per RCP 2.6). This surge is attributed to the high rainfall prediction for that particular year. Such variations in EIR highlight the sensitivity of malaria transmission to climate conditions, especially rainfall patterns.

In conclusion, the study’s key results demonstrate a decline in malaria transmission, as indicated by the decreasing EIR, which can be associated with global warming and changes in temperature and rainfall. However, it is crucial to recognize that while this decrease in malaria risk is positive, the alterations in climate have broader implications for various aspects of life and ecosystems. Additionally, the prediction of a surge in EIR in 2027 and 2024 emphasizes the importance of monitoring and understanding the complex relationship between climate and malaria transmission for effective public health planning and interventions.

### Monthly trends

**Fig. 3:**
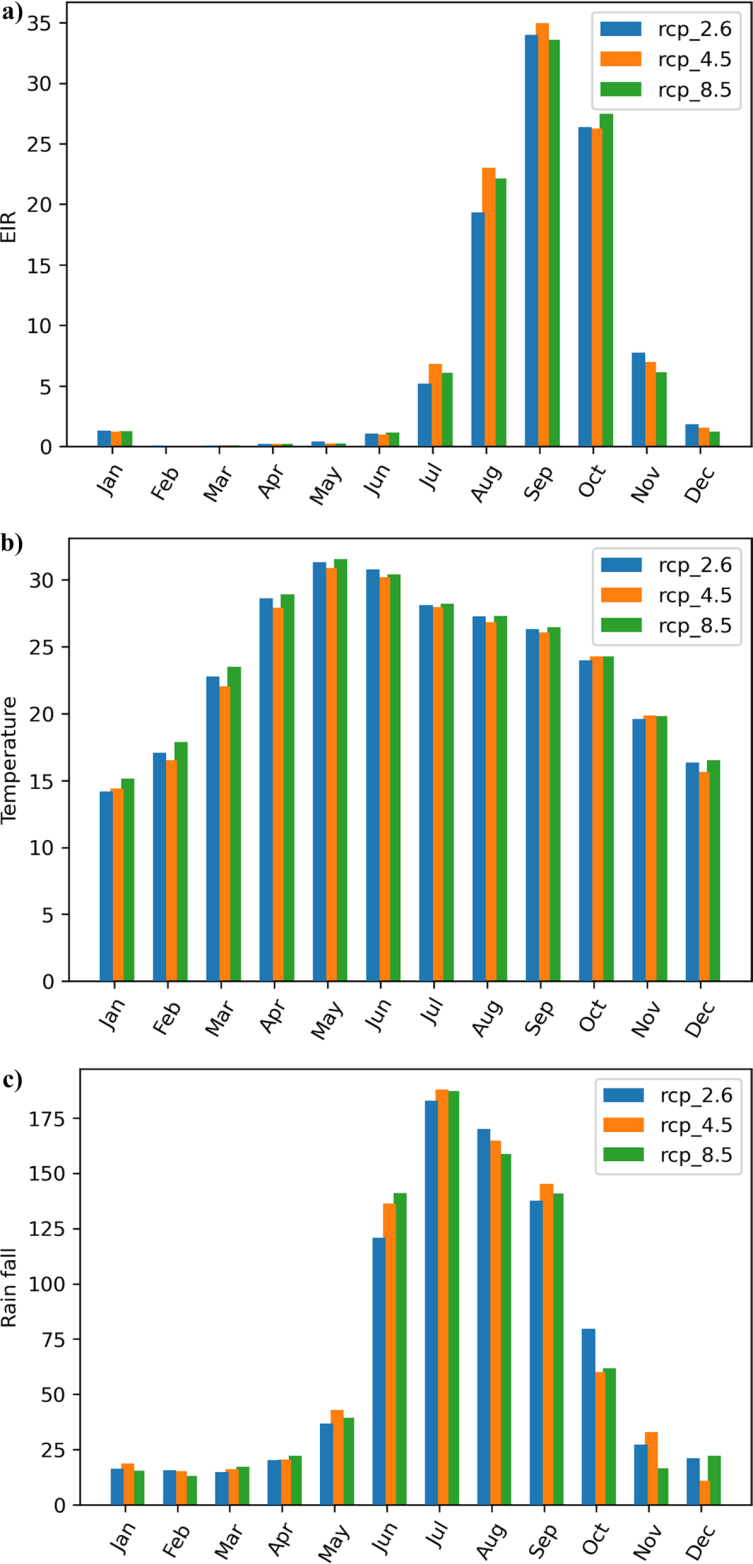
Monthly variation EIR, temperature and precipitation

The monthly trends reveal that the middle of the monsoon season to the post-monsoon season (September to November) are the months with the highest malaria transmissions. During this period, the infectious mosquito bites per person per month (as shown in Fig. 3a) can reach up to 30–35. These months are characterized by average temperatures of approximately 25–28 °C (Fig. 3b) and sufficient rainfall (Fig. 3c), creating conditions conducive to malaria prevalence. While rainfall plays a significant role in malaria transmission, it is not the sole determining factor. The model considers thresholds for both rainfall (7–8 mm/day) and temperatures (15–30 °C), indicating that malaria development exhibits a non-linear relationship with climatic conditions. In Fig. 3, the blue colour represents the base RCP 2.6 emission mean, orange belongs to RCP 4.5, and green represents RCP 8.5.

As depicted in Fig. 3b and c, the most favourable conditions for malaria transmission occur in September, with the best combination of rainfall and temperatures being around 130 mm/month and 26 °C, respectively. On the other hand, the EIR is nearly zero during the months of January to March. During the hot monsoon days and cold seasons in India, the malarial risk is very low due to extreme cold and hot temperatures.

In summary, the study highlights that the months from the middle of the monsoon season to the post-monsoon season are the peak malaria transmission months, characterized by suitable temperatures and rainfall and this is well established fact from historical observations. However, these trends will increase with RCP in future scenarios. These conditions promote mosquito breeding and transmission. Conversely, the risk of malaria is significantly reduced during the hot monsoon and winter seasons due to extreme temperatures. The model used in the study considers both rainfall and temperature thresholds, illustrating the non-linear nature of malaria development in response to climatic factors.

From Fig. 3a, the highest increment in EIR is observed in August month in the RCP 4.5 case and a slight increment in July with RCP 4.5 and October with RCP 8.5.

### Spatial trends with least emission case RCP 2.6

**Fig. 4:**
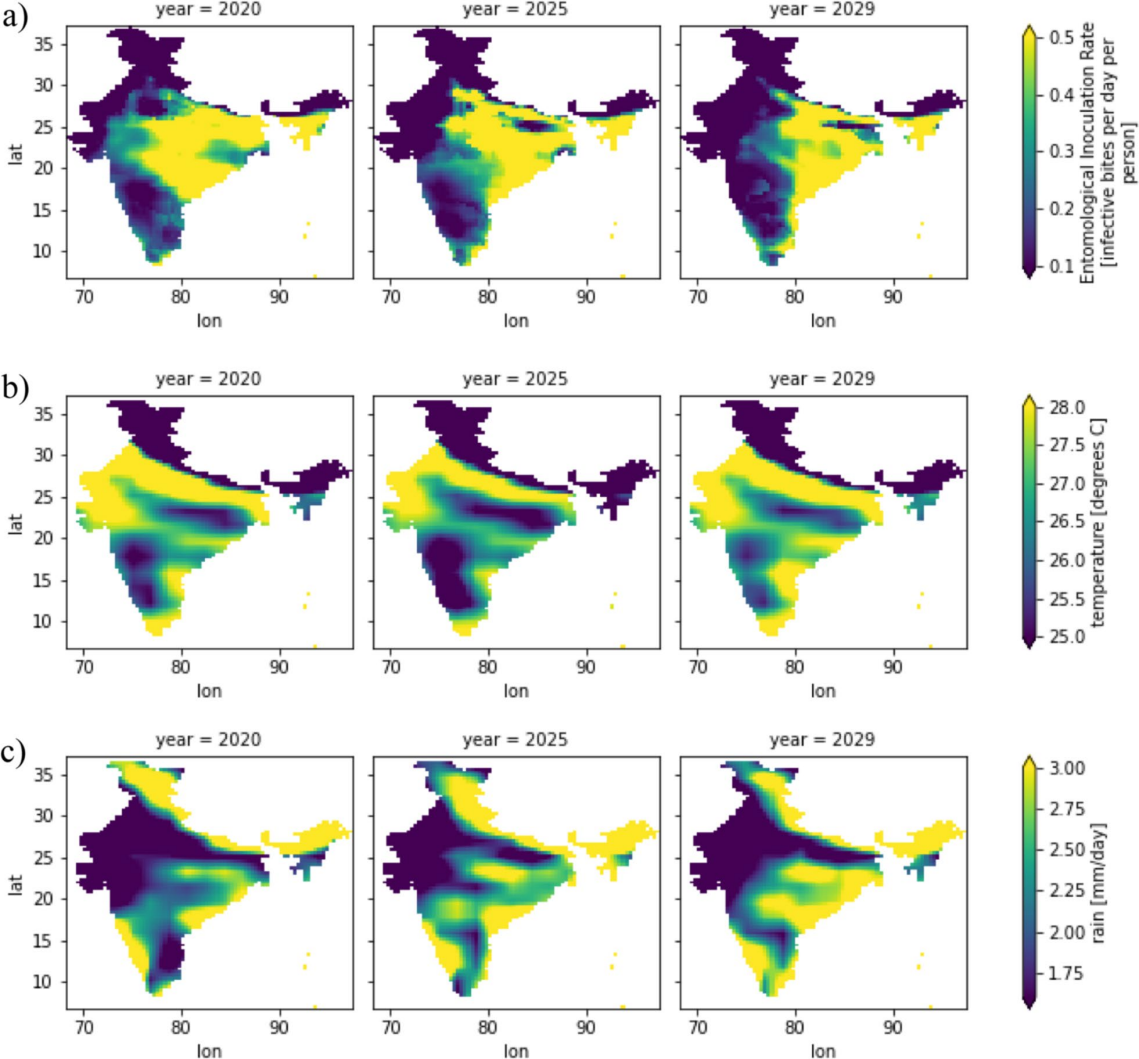
Spatial trends of EIR (83 × 81 pixels and each pixel is 36 km.^2^), temperature and precipitation of 2020, 2025 and 2029

From 2020 to 2029, the spatial patterns of malaria transmission will fallow consistent follow trends. The regions with the highest values of Entomological Inoculation Rate (EIR) are the east coast of India and the Indo-Gangetic Plains (IGP). As shown in Fig. 4, these areas exhibit a habitable environment characterized by high rainfall and optimum temperatures. The air temperatures in these regions range between 25 and 30 °C, while the average rainfall is around 2.5 mm per day.

Conversely, Jammu and Kashmir has the lowest EIR due to extremely cold temperatures below 25 °C, despite receiving sufficient rainfall of more than 2 mm per day. Similarly, the northwestern regions, particularly Rajasthan, also have a low EIR due to extremely hot temperatures exceeding 25 °C and very minimal rainfall, less than 1.8 mm per day.

As observed in Fig. 4, the overall magnitude of EIR has been decreasing from 2020 to 2029. However, there has been a spatial shift of EIR from the central part of India towards the east coast. This shift can be attributed to the higher precipitation rates in the east-coast region, where an increment of approximately 0.5 mm/day in rainfall has been observed.

Meanwhile, areas with extremely cold temperatures, like Jammu and Kashmir, and regions experiencing extremely hot temperatures and minimal rainfall, such as Rajasthan, exhibit lower malaria risk. The overall trend indicates a decline in EIR, but the shift of transmission towards the east coast of India is likely influenced by the increasing rainfall in that region.

### Spatial trends comparison between RCP 2.6, 4.5 and 8.5

**Fig. 5:**
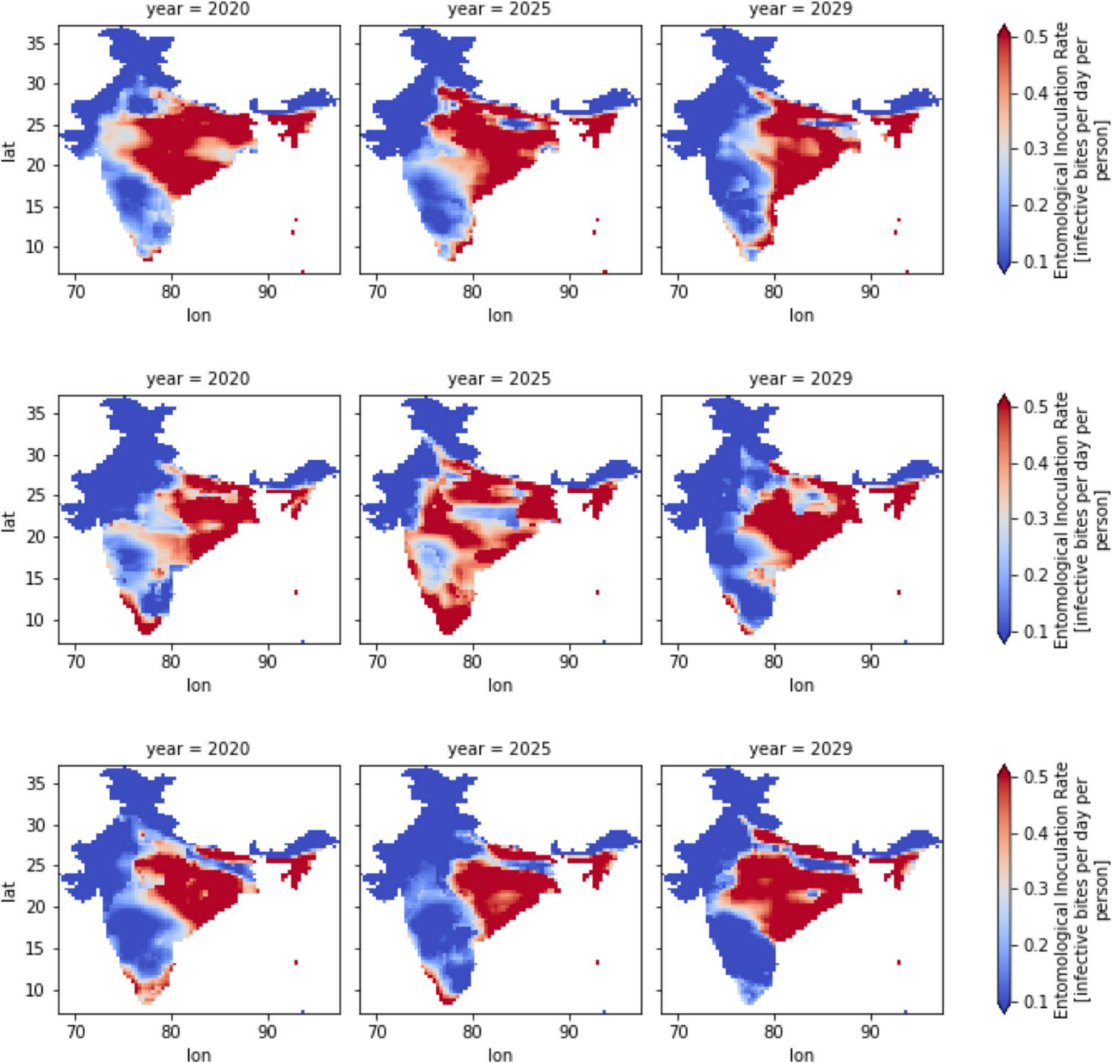
Comparison of spatial trends of EIR (83 × 81 pixels and each pixel is 36 km^2^), of RCP 2.6, 4.5 and 8.5

**Fig. 6:**
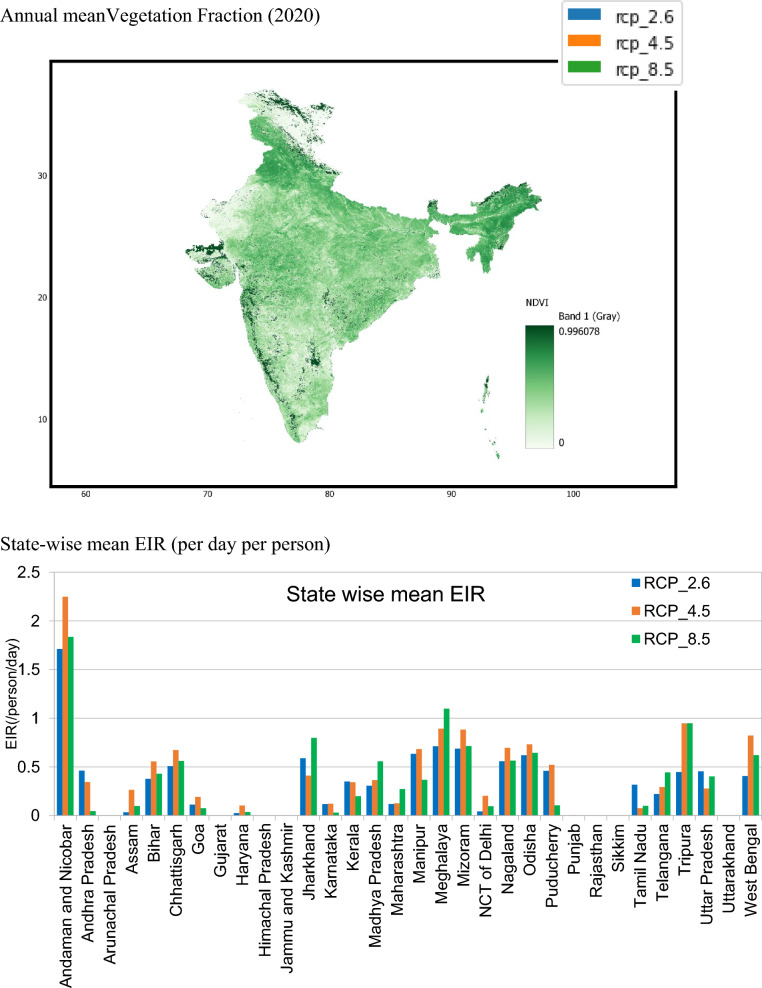
State-wise distribution of EIR by the year 2029 with RCP 2.6, 4.5 and 8.5

Figure 5a–c represent annual spatial EIR over India. As discussed earlier, the annual mean of EIR is decreasing, but from Fig. 5a–c, spatial EIR has changed significantly over India. In all three potential scenarios, the eastern coast of India is more vulnerable to malaria risk. Specifically, in Fig. 5b, the year 2025 of the RCP 4.5 scenario shows a slight increase in risk throughout southern India. A high risk is observed over northeast India, which is witnessed with all RCP cases. In the RCP 2.6 case, the magnitude of EIR is more than 0.5 (per person per day), and it is about 0.4 (per person per day), with RCP 4.5 and again more than 0.5 with RCP 8.5. However, the mean of EIR/year is higher RCP 8.5 and followed by RCP 4.5 and least with RCP 2.6. The states with high vegetation rates, such as Andaman and Nicobar, Meghalaya, and Tripura, showed a high EIR. The east-cost states such as West Bengal, Andhra Pradesh, and Tamilnadu showed a moderate risk. The west coast and northwestern regions showed very low risk. In Fig. 6, the colour pattern is the same as in Fig. 3; thus, blue represents RCP 2.6, orange represents RCP 4.5, and green represents 8.5. Thus, from Fig. 6, most variations are observed with RCP 4.5, such as Andaman and Nicobar, Tripura, and West Bengal. The RCP 8.5 scenario affected some states, such as Jharkhand, Madya Pradesh, Meghalaya, and Telangana.

### Regression model to find risk in coming years

**Fig. 7:**
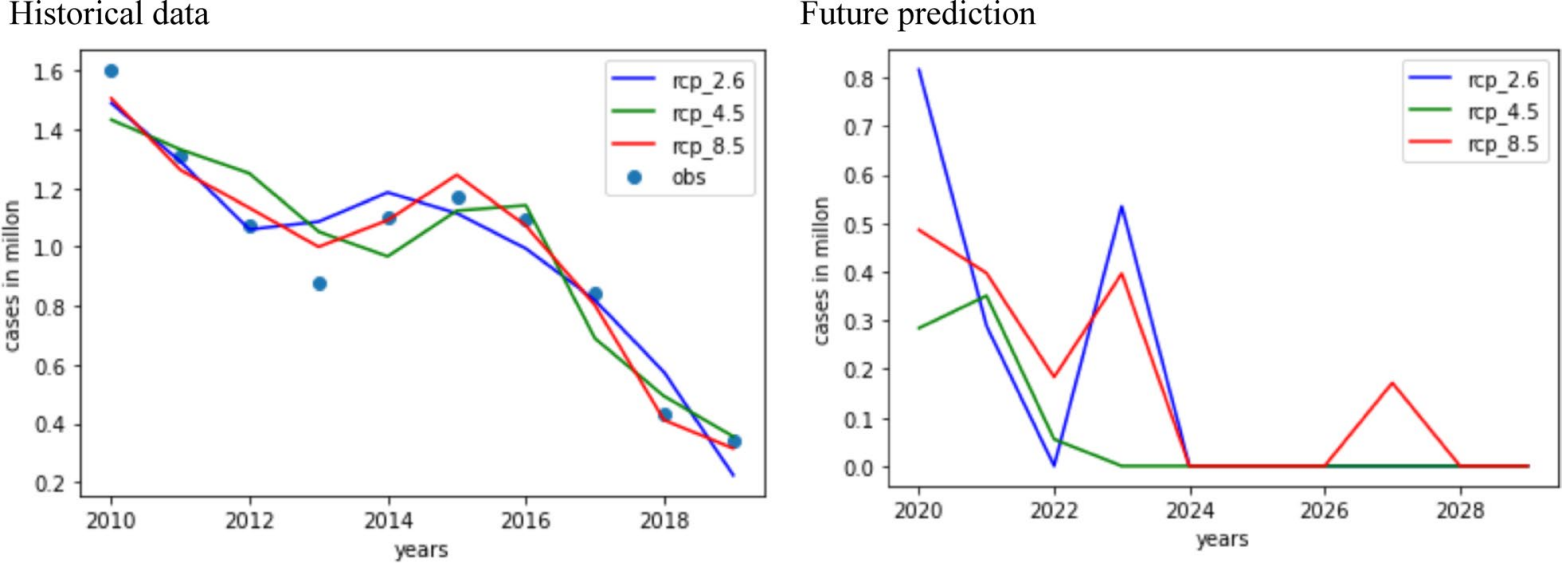
Regression models to historical data and future prediction

From Fig. 7, the number of cases has decreased almost 10 times in the last decade (i.e. 1.4 million to 0.4 million). This drastic decrease is due to the Indian government’s initiative to control malaria. The Government of India has set a national target of eliminating malaria by 2030. The goal is to have zero indigenous cases of malaria throughout the country by then and to maintain malaria-free status in areas where transmission has been interrupted. The National Framework for Malaria Elimination (NFME) 2016–2030 was launched in February 2016 and aligns with the World Health Organization’s (WHO) Global Technical Strategy (GTS) for Malaria 2016–2030. The interesting fact is that even though the regression model showed (Fig. 7b) that malaria will be eliminated by 2030 still, the point to be noted is that the year 2027 is risky even with this elimination rate. The sharp surge in EIR of about 140 bites/person/year in 2027 may have an impact on the control programme. This research provides policy-makers with insight into the strict implementation of the malaria elimination strategy [22]. It is quite interesting that a similar time-series analysis in recent research [23] also showed zero incidences can be achieved within 2030. Thus, this analysis will be an add-on and vision to achieve zero malaria incidence targets.

### Validation

**Fig. 8:**
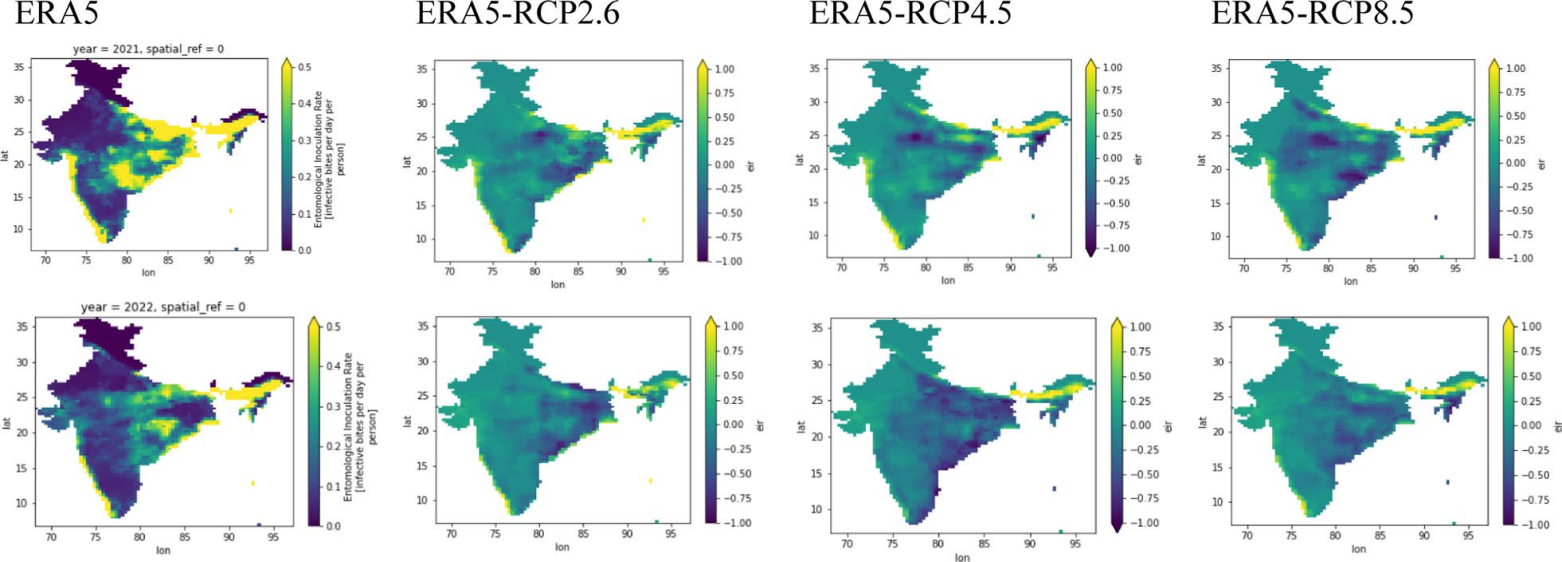
Validation of EIR (83 × 81 pixels and each pixel is 36 km^2^), from real-time reanalysis data and future case scenarios

The model is based on physical equations and climatological parameters. Since it is an estimation of spatial EIR of each day at each grid level. Thus, to calibrate the model, the true climatological parameters from reanalysis data (ERA5) for 2021 and 2022 are taken, and simulations are carried out with these data sets. From Fig. 8, EIR was computed with ERA5 data and EIR from RCP 2.6, RCP 4.5 and RCP 8.5 are subtracted from the EIR of ERA5 data. The maximum difference is ranged from -0.2 to 0.2 over the domain. The futuristic scenarios are underestimated in the East Coast region and slightly overestimated in the northeastern and at some parts of the West Coast.

## Discussion

This study’s main objective is to project malaria’s probabilistic EIR under conditions involving different possible emissions and corresponding radiative forcing. The prominent findings align well with previous research [20, 24].

Earlier studies like that of [20] have quantified malaria risk using different projections (RCP 2.6, 4.5, 8.5), and their results are mostly consistent, even when using RCP 2.5. However, it presents some noteworthy outcomes, indicating a decline in malaria risk.

Some of the conclusions suggest that the future climate may become less suitable for malaria vectors, which aligns well with other recent studies like those by [25, 26] and the study conducted by Sharma et al*.* [27] that demonstrated a decline in malaria cases over the past decade. Nevertheless, the present findings offer more consistency in terms of monthly variations, highlighting the post-monsoon period as the most suitable habitat for malaria transmission across India, consistent with [28–31].

Moreover, the spatial analysis also shows strong agreement with a recent study by [20, 24], revealing high malaria risk in the east coast of India, with an average EIR exceeding > 0.4 bites per person per day. The shift of malaria risk towards eastern India is also predicted in earlier research, such as that by [12, 20].

There are a few limitations in implementing the model.

1. The noteworthy point is that our data is rescaled and taken from CMIP5 models over the domain, so spatial bias [18] might exist.
2. Here, an interesting point is some prominent research [32, 33] corrected the biases and these procedures can be implemented in future research and can do sensitivity analysis.
3. The model was only well calibrated for *An. culicifacies*, the true EIR might be more when including other vectors.
4. More spatial accurate models and more accurate population density models can be implemented.
5. Parametrization schemes to local topography and Indian climatic conditions can be modelled.
6. The initiative of the government such as zero incidence policies will reduce malaria transmission significantly but it will not be reflected in model.

Along with these limitations, the present study exclusively focusing on the *Anopheles* species present in India. However, the model was originally calibrated for the African region where *Anopheles gambiae* is the prime vector, but the model still performed well at that site due to the environment’s suitability for that particular vector.

## Conclusions

The observed decline in malaria risk is a positive development, but it is essential to exercise caution, especially in areas with heavy rainfall. There is room to improve the model based on other CMIP 5 datasets with better spatial accuracy. The inclusion of the future projection of the population dataset instead of census 2011 may improve model accuracy. Since it is a mechanistic model and mostly relies on temperature and precipitation, the results are limited to climatological conditions only. However, in real-time scenarios, there may be different spatial patterns based on other factors, such as low sanitation access areas with greater accumulation and states that poorly take care of mosquito mitigation strategies. Thus, human intervention may mostly modify results. Furthermore, observational studies are required to cross-validate and improve the models according to needs. In all odds, this research provides valuable insights for policy-makers, highlighting the need to implement future strategies to mitigate malaria risk effectively. By utilizing these findings, appropriate measures can be taken to combat the threat posed by malaria and protect public health.

## Data Availability

The data collected and used for the analysis are freely available and reproducible. The CMIP data is collected from Copernicus ECMWF data portal (https://cds.climate.copernicus.eu/cdsapp#!/dataset/projections-cmip5-daily-single-levels). The population density is taken from Indian Censes data (https://censusindia.gov.in/census.website/data/population-finder).
